# Fish personality: meta-theoretical issues, personality dimensions, and applications to neuroscience and psychopathology

**DOI:** 10.1017/pen.2023.3

**Published:** 2023-10-19

**Authors:** Ana Carolina Luchiari, Caio Maximino

**Affiliations:** 1 Department of Physiology & Behavior, Universidade Federal do Rio Grande do Norte, Natal, Brazil; 2 Laboratório de Neurociências e Comportamento, Instituto de Estudos em Saúde e Biológicas, Universidade Federal do Sul e Sudeste do Pará, Marabá, Brazil

**Keywords:** Behavioral syndromes, Evolution of personality, Fish personality, Neural bases of personality

## Abstract

While the field of personality neuroscience has extensively focused on humans and, in a few cases, primates and rodents, a wide range of research on fish personality has emerged in the last decades. This research is focused mainly on the ecological and evolutionary causes of individual differences and also aimed less extensively at proximal mechanisms (e.g., neurochemistry or genetics). We argue that, if consistent and intentional work is made to solve some of the meta-theoretical issues of personality research both on fish and mammals, fish personality research can lead to important advances in personality neuroscience as a whole. The five dimensions of personality in fish (shyness-boldness, exploration-avoidance, activity, aggressiveness, and sociability) need to be translated into models that explicitly recognize the impacts of personality in psychopathology, synergizing research on fish as model organisms in experimental psychopathology, personality neuroscience, and ecological-ethological approaches to the evolutionary underpinnings of personality to produce a powerful framework to understand individual differences.

The landscape of personality research has been dominated by work with humans; however, in order to understand the neurobehavioral bases of personality, the use of model organisms is a fundamental step. While most researchers are likely to look into research on closely related mammals, including primates, an expanding field has been the study of personality in fish. Since the seminal work of Huntingford (1976) on individual differences in sticklebacks, the field of fish personality has steadily grown, now involving a wide array of species, tests, and empirical data on personality dimensions (Conrad, Weinersmith, Brodin, Saltz, & Sih, 2011; Réale, Reader, Sol, McDougall & Dingemanse, 2007). The interest in using fish sprung not only from the relatively ease of using small teleosts as laboratory model organisms (Stewart et al., 2015) but also due to the tradition of fish research in ethology. Combining approaches from both ethology and neuroscience has the potential to transform the field of personality neuroscience, but, as will be discussed in the present article, many issues still make it difficult to compare data from both fields.

The present article is a discussion of the implications of fish personality research to personality neuroscience in general. We begin by presenting some of the current conundrums in fish personality research, focusing on a meta-theoretical framework proposed by Jana Uher (Uher, 2008, 2011, 2013, 2015; Uher, Werner & Gosselt, 2013). In Section 2, we focus on the empirical research that defined a five-dimensional model of personality across fish species, commenting on some of the findings on proximal mechanisms that might be relevant to personality neuroscience. We close the article by presenting implications of fish personality research to experimental psychopathology, using the relationship between personality and individual differences in anxiety-like behavior in zebrafish as a case study.

## 1. Current issues in fish personality research

One important issue in the field of animal personality in general is how one defines *personality*. This question is important because it represents a theoretical constraint on how to measure personality in non-linguistic species, as well as in non-mammals. Theory always guides judgments about what counts as data in a given field, and how these data can be analyzed and interpreted (Cervone, Shadel & Jencius, 2001; Køppe, 2012; Uher, 2015); theories of personality always contain not only a theory of individual differences but also a theory of how to assess these differences (at least implicitly). Thus, theories of personality always should ask: which kinds of phenomena should be considered in assessing individual differences? Should the focus be on behavior, emotionality, cognition, motivation, or something else? What kind of differences are important: between-subject differences, consistency in these differences, or within-subject differences?

Closely related to these issues is the methodological question of the unit of analysis: while exploration of differences between populations is fundamental to identify what is specific to each individual (Uher, 2013), in the end the question of individual differences is also targeted at exploring individual consistency (Allport & Allport, 1921; Uher, 2011). Thus, one important methodological question in the field of personality research is how to define patterns of phenomena (e.g., behaviors) both at the population and individual levels (Carter, Feeney, Marshall, Cowlishaw & Heinsohn, 2013; Sánchez-Tójar, Moiron & Niemelä, 2022). It has been argued (Kaiser & Müller, 2021) that, in order to speak of non-human personality, population-level and between-individual differences are necessary to identify broader patterns, but these data must in turn be able to define a behavioral trait in individual animals: the individual must behave differently than others (i.e., show individual differences); these behavioral differences must be stable over a certain time (i.e., temporal stability), and they must be consistent in different contexts (contextual consistency) (Castanheira, Herrera, Costas, Conceição & Martins, 2013; Kaiser & Müller, 2021).

An example of research that demonstrated that a specific stability of correlations between behavioral variables at the population levels did not hold at the individual level is that made by Lee and Bereijikian (2008) on brown rockfish (*Sebastes auriculatus*): positive correlations were found at the population level between predator inspection and feeding in absence and presence of predators, and these correlations still held 10 days later. While this would usually be interpreted as a sign of a “personality,” the individuals’ rank order along the different behavioral variables changed considerably. This is not an indication of individual behavioral phenotype and, as a result, not consistent with the term “personality” (Uher, 2011).

Most of the research that is discussed in the present article is relevant to understanding fish personality even though individual consistencies are not always evaluated. Especially when the final aim is to describe proximal causes and mechanisms, most studies on fish personality measure differences at the population level, without looking at individual differences and consistencies (Conrad et al., 2011; Toms, Echevarria & Jouandot, 2010). As a result, while certainly relevant as hypothetical-generating devices, these studies do not target personality *per se*.

Finally, the question of *mechanism* is highly important to the field of personality. The field of animal personality can be approached both from the point of view of distal (e.g., evolutionary) mechanisms and from the point of view of proximal mechanisms (e.g., biological basis) (Weiss, 2018). However, the particular mechanisms that are looked for depend heavily on the conceptual and methodological issues (Uher, 2015).

One of the implicit decisions that is apparent in fish personality research is the assumption that the traits or dimensions that are observed are *evolved*. As remarked above, the field of fish personality research blossomed in the hands of researchers who were trying to understand the distal mechanisms of personality. Following the dominant *adaptationist* paradigm, this research usually understands personality traits as adaptive and “selected for,” and part of the research in the field revolves around trying to find the selective pressures that led to the evolution of the specific trait or traits (Schuett, Tregenza & Dall, 2010; Sih, Bell, & Johnson, 2004). Again, variation across personality dimensions is usually assessed as behavioral variation between *populations* and not as variation between individuals (i.e., within-population variability) (MacKay & Haskell, 2015). While such variation is certainly important if we are to understand the evolutionary bases of personality, *within-individual* consistency is necessary to define a behavioral trait in individual animals, and individual variation is important to understand the proximal mechanisms which are relevant to personality neuroscience (MacKay & Haskell, 2015; Pervin, 1994; Uher, 2011).

A closely related question is that of selection towards one or more optimal strategies (Carter et al., 2013): if selection acts towards optimality, how is variation in animal behavior maintained? However, most studies on fish personality so far have not explicitly tested adaptationist hypotheses that are related to the specific contexts in which species-specific selective pressures are taking place, instead looking for pressures that appear to be universal. This, of course, facilitates a comparative framework, but it also assumes the universality of traits. Uher (2008) proposed that assuming a behavioral repertoire approach in non-human personality research will necessitate explicitly recognizing that the dimensions or traits that will be investigated are not *necessarily* universal nor unique; in fact, because “all species have a phylogenetic history and show adaptations to a particular ecological niche, most species exhibit both universal *and* unique trait dimensions in their personality structure” (Uher, 2008, p. 479).

These assumptions, of course, determine which behaviors will be selected as endpoints in personality research. Uher (2008) identifies two rationales for selecting endpoints: a *bottom-up approach*, which usually starts from the exhaustive measurement and cataloging of observable behavior in a population, followed by subsequent theorizing and analysis of Tinbergen’s four questions (function, evolution, causation, and development), and a *top-down approach*, which usually starts from theories of personality that were created based mainly on human data (e.g., the Reinforcement Sensitivity Theory (RST); Corr, 2004) and attempts to apply it to other species. In fish personality research, the bottom-up approach is much more common (Toms et al., 2010) – in fact, the majority of non-human personality studies which claim the *shyness-boldness dimension* that will be discussed below were made first in fish species (Conrad et al., 2011; Sih, Bell, & Johnson, 2004; Sih, Bell, Johnson, et al., 2004). However, some attempts have been made to “map” the personality dimensions empirically described in fish to other theories, such as RST (Maximino et al., 2012).

## 2. Dimensionality of personality in fish

Many authors have pointed out that the lack of consensus on measurement reflects the lack of consensus on the definitions of specific dimensions or traits in non-human personality (Carter et al., 2013; Toms et al., 2010). This is a meta-theoretical issue as well (Uher, 2013, 2015): should a continuum such as shyness-boldness be considered as a *dimension*, with possible values falling all over the continuum, or as a *trait*, with possible values falling on limited portions of the continuum? While partially solvable by empirical research using appropriate statistical methods (Toms & Echevarria, 2014), this meta-theoretical issue is commonly left implicit in the field of fish personality research. Moreover, researchers of “(non-human) animal personality” usually study specific personality traits or dimensions, such as shyness-boldness, sociability, or aggressiveness, rather than personalities in general (Kaiser & Müller, 2021). Nonetheless, the use of terms such as “personality dimensions” implies the existence of a “global” personality that is decomposed into these dimensions (Kaiser & Müller, 2021).

This problem gave rise to differences in terminology in the field. The terms “personality,” “temperament,” and “behavioral syndrome” are sometimes treated as interchangeable, leading to conceptual confusion. We follow Kaiser and Müller (2021) by defining *personality* as the individual variation, consistent across time and contexts, across all possible dimensions/axes. A personality *dimension* is understood as a single axis (e.g., aggressiveness or shyness-boldness) in which population-level variation can be mapped. Finally, we apply the term “behavioral syndrome,” introduced by Alison Bell (Bell, 2007; Sih, Bell, & Johnson, 2004; Sih, Bell, Johnson, et al., 2004), in the same sense that was proposed by MacKay and Haskell (2015) as the link between “two or more dimensions across a population” (p. 41). Thus, a behavioral syndrome is a suite of correlated behaviors across situations and contexts which exists within a population (Conrad et al., 2011; Sih, Bell, & Johnson, 2004; Sih, Bell, Johnson, et al., 2004). For example, boldness-aggression syndromes have been shown within some populations of zebrafish or sticklebacks (*Gasterosteus aculeatus*), but not in other populations (Bell, 2005; Martins & Bhat, 2014; Roy & Bhat, 2018a, 2018b). This is a necessary definition to allow for comparability of research made by ethologists and animal behaviorists with that made by psychologists, who would certainly not consider within-population variation in a single dimension as evidence for a “personality” (Pervin, 1994; Uher, 2011). Mere evidence of within-population correlations between dimensions, without evidence of stable within-individual correlations, is not sufficient to affirm a “personality” (see Uher, 2011, for a thorough discussion of this topic).

**Figure 1.**
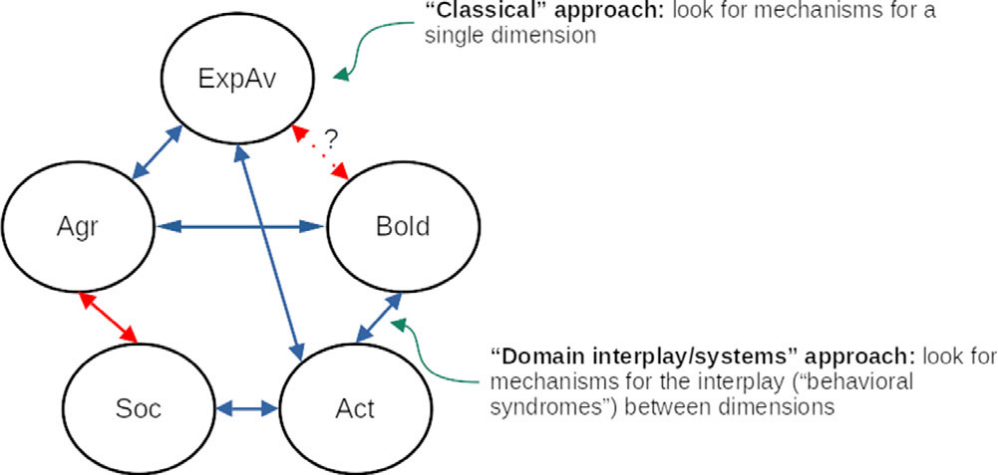
Five-dimensional model of fish personality and observed or inferred correlations between dimensions. Blue arrows denote positive correlations, while red arrows denote negative correlations. The “classical” approach in fish personality research is to look for mechanisms in single dimensions, while a “system” approach involves looking for mechanisms for the interplay between dimensions (i.e., for *behavioral syndromes*). Abbreviations: ExpAv: Exploration-avoidance; Bold: Shyness-boldness; Act: Activity; Soc: Sociability; Agr: Aggressiveness.

In this review, we follow the framework by Réale et al. (2007) to describe the behavioral dimensions of fish personality: sociability, aggressiveness, exploration-avoidance, activity, and shyness-boldness (Fig. 1); however, we also echo the concerns of Conrad et al. (2011) that simply referring to the term used for a specific dimension is not enough, and grasping the full meaning of that dimension/trait is only possible by considering the context and methods of each study. In what follows, we briefly define these dimensions, describe some of the behavioral tests that are used to assess them (Table 1), and then present some results on eco-ethological research and on mechanistic research that are likely to be relevant to the field of personality neuroscience.

**Table 1.** Summary of behavioral tests commonly used to measure each dimension of fish personality. Note that some tests can measure more than one dimension

Dimension	Behavioral tests	Example of operational definition	Neurotransmitter system involved
Shyness-boldness (Bold)	Responses to predators	Avoidance or inspection of the predator	Serotonin
		Time spent foraging under risk	
	Response to threatening stimuli	Frequency of freezing	
		Latency to feed after the introduction of stimulus	
Exploration-avoidance (ExpAv)	Emergence test	Latency to emerge from a refuge	Serotonin, dopamine, histamine, glucocorticoids
	Novel object test	Latency to approach a novel object	
		Proportion of time spent in contact with the object	
	Light/dark test	Proportion of time spent in the non-preferred compartment (e.g., black in adult zebrafish)	
	Novel tank test	Proportion of time spent in the bottom third of the tank	
Activity (Act)	Novel tank test	Distance covered	
		Swimming speed	
		Squares crossed	
	Other tests (Virtually every behavioral test in other dimensions can also incorporate activity measures)	Distance covered	
		Swimming speed	
		Squares crossed	
Sociability (Soc)	Social preference test	Time spent near conspecific	Sexual steroids, nonapeptides, dopamine
	Social novelty test	Time spent near novel conspecific	
	Shoaling	Inter-fish distance	
	Conditional approach	Time spent inspecting predator when conspecific is present	
Aggressiveness (Agr)	Mirror test	Aggressive display or contact	Serotonin, dopamine, histamine
	Aggressive encounters	Aggressive display or contact	

### 2.1 Boldness-shyness

Réale et al. (2007) define “boldness” as behavior in situations perceived as dangerous, excluding reactions to novelty. This definition was based on studies that were unable to find correlations between responses to threatening novel stimuli with responses to non-threatening novel stimuli (e.g., food in Coleman & Wilson, 1998). However, this definition is also over-reductive, as it ignores the possibility that novelty is also threatening (Kaiser & Müller, 2021). In fact, there is a long tradition of research on exploratory behavior in rats that recognizes that at least two factors underline this behavior: an approach motivation (“curiosity” or “exploration”) and an avoidance motivation (“fear”) (Hughes, 1997; Montgomery, 1955; Montgomery & Monkman, 1955; Russell, 1973). These two-factor theories were also highly influential in motivating theoretical models, such as RST (Corr, 2004; Corr & McNaughton, 2012). In contrast to Réale et al. (2007), Toms et al. (2010) argued that tests involving novelty are best suited for capturing a personality dimension of “shyness-boldness,” while measuring risk-taking in the presence of predators would capture situationally defined reactions but not necessarily boldness.

To further complicate the issue, boldness is commonly assessed using emergence tests, in which the key measure is the latency to leave a “safe” chamber and enter a “risky” arena, which nonetheless has no predators or other clearly threatening stimuli. In these situations, the “risky” arena represents a potentially threatening place, but it is not clear how this maps to Réale et al.’s (2007) definition of boldness. In some studies (e.g., Ferrari et al., 2016), traits defined as “boldness” using variations of the emergence test have been used to define “coping styles,” with bold individuals described as “proactive” and shy individuals described as “reactive.” However, definitions of coping commonly used in fish research are one-dimensional (Koolhaas et al., 1999), and coping is highly plastic (Øverli et al., 2007). Another source of confusion is that some researchers (e.g., Sih, Bell, & Johnson, 2004) treat coping as an “axis” while simultaneously defining proactive individuals as “both aggressive and bold,” suggesting a confusion between a personality dimension and a behavioral syndrome.

Even if only studies of behavior under distal or proximal threat are considered, a shyness-boldness dimension has been documented in a variety of fish species. Studies in sticklebacks (*Gasterosteus aculeatus*) show that the ontogenetic stability of boldness is variable between populations (Bell & Stamps, 2004) and that boldness varies across habitats as a function of predation risk (Álvarez & Bell, 2007). These results were conceptually replicated in populations of poeciliids (*B. episcopi*) (Brown & Braithwaite, 2004; Brown, Jones & Braithwaite, 2005; Brown, Jones & Braithwaite, 2007) and zebrafish (*Danio rerio*) (Roy & Bhat, 2018b, 2018a). In all cases, individuals from habitats with higher predation risk are bolder, illustrating the “paradox of risk allocation” (Ferrari, Sih & Chivers, 2009).

Boldness has been considered a trait with high heritability in fishes (Brown, Burgess & Braithwaite, 2007; Ferrari et al., 2016; Mazué, Dechaume-Moncharmont & Godin, 2015) as in other animals, although the environment plays an important part in shaping individual levels of boldness (Polverino, Cigliano, Nakayama & Mehner, 2016; Stamps & Groothuis, 2010). Longitudinal studies followed the ontogenesis of this trait in search of some temporal consistency, but it is worth noting that behavioral plasticity figures as a valuable element for individual fitness. For example, sex, age, body size, and hierarchical status are known factors that affect boldness (Castanheira et al., 2016; King, Fürtbauer, Mamuneas, James & Manica, 2013; Philip, Dellinger & Benhaïm, 2022). Moreover, several environmental factors experienced during early development, such as food availability, pH variation, and temperature, affect boldness later in life (Bell & Stamps, 2004; Stamps & Groothuis, 2010; Stamps & Groothuis, 2010).

The shyness-boldness continuum is sometimes correlated with aggressiveness, forming a boldness-aggression syndrome. In Fig. 1, this is represented by individuals A and B showing higher levels of both boldness and aggressiveness, and individual C showing low levels of both boldness and aggressiveness. The stability of these associations seems to depend on the population. For example, studying zebrafish populations collected in the wild, Martins and Bhat (2014) found major population differences in the levels of aggression and boldness (using a simulated predator attack), but found correlations between aggression and boldness in only one of the five populations. In sticklebacks, positive correlations between aggression and boldness were also described. Associations at the population level were found by Bell, Henderson and Huntingford. (2010), who found that sticklebacks derived from populations from high predation sites had higher boldness (measured by predator inspection) and conspecific aggression. Bell and Sih (2007) found that sticklebacks which were exposed to predation show correlations between boldness (measured by a simulated strike assay) and aggression, while animals which were not experimentally exposed to aggression did not show these correlations. Roy and Bhat (2018a) also found in zebrafish that predation levels are related to associations between boldness (using a predator inspection assay) and other dimensions; a negative relationship was found between activity and boldness only within two low-predation populations. In general, then, associations that are found at the population level do not always translate to *individual* differences and therefore might be more related to population-level selective pressures than individual variability.

Few attempts have been made to understand the neurobiological bases of this boldness-aggression syndrome. In both the fish and the mammalian literature, aggressiveness and responses to distal or proximal threat are both negatively related to serotonin levels (Filby, Paull, Hickmore, & Tyler, 2010; Graeff, 2004; Lima-Maximino et al., 2020; Olivier, 2004; Paul, Johnson, Shekhar & Lowry, 2014; Paul & Lowry, 2013). It would not be surprising, then, to find that the boldness-aggression syndrome is related to this monoamine. Bell, Backström, Huntingford, Pottinger and Winberg (2007) assessed the levels of monoamine neurotransmitters in different regions of the stickleback brain when exposed to an unfamiliar conspecific or a predator; hypothalamic serotonin was negatively correlated with frequency of attacking a conspecific and positively associated with predator inspection, suggesting this neurotransmitter as a potential link between aggression and boldness.

### 2.2 Exploration-avoidance

The exploration-avoidance dimension includes behaviors that involve individual differences in willingness to investigate novel environments, food items, or objects (Conrad et al., 2011). The most commonly used tests for anxiety-like behavior in zebrafish rely on exploratory behavior under conditions of novelty, but not explicit threat (Kalueff et al., 2016; Kysil et al., 2017; Maximino et al., 2010, 2012) – and, while the shyness-boldness dimension should conceptually include behavior in anxiety-inducing environments (Kaiser & Müller, 2021; Maximino et al., 2012), by the definition of shyness-boldness and exploration-avoidance used in most of fish research these tests should be considered indexes of the latter. Typical measures of the exploration-avoidance dimension in fish include the animal’s latency to explore a novel arena or to emerge from a shelter (“emergence tests”), the latency or time spent exploring a novel object, or the latency to consume a novel food (Conrad et al., 2011; Réale et al., 2007). It bears repeating that a lot of research using these tests talks of boldness instead of exploration-avoidance, leading to great confusion; however, to be distinguished from shyness-boldness, exploration-avoidance should be assessed in the absence of threatening stimuli other than novelty itself (Conrad et al., 2011; Réale et al., 2007).

Despite these different definitions, the exploration-avoidance continuum has also been linked to neophilia or neophobia. This is important in the context of two-factor theories of exploratory behavior (Hughes, 1997; Montgomery, 1955; Montgomery & Monkman, 1955; Russell, 1973) that suggest that, in novel environments, exploration is controlled both by an approach motivation (“curiosity” or “exploration”) and an avoidance motivation (“fear”). From an ecological point of view, exploratory tendency has been exploited as a sign of fitness: golden shiners (*Notemigonus crysoleucas*) that are quicker to navigate through a novel tank were also more likely to be group leaders in a shoal (Leblond & Reebs, 2006). Likewise, Nomakuchi, Park and Bell (2009) showed that sticklebacks with higher exploration were also more likely to learn a maze through social learning. These unexpected correlations between exploration-avoidance and sociality still need to be more deeply investigated to understand whether low exploration/low sociality individuals exist within populations, which could point to different neurobiological bases for high exploration and low exploration.

The ontogenesis of the exploration-avoidance dimension has been more thoroughly investigated than boldness. Using emergence tests, some authors observed within-population differences in exploration-avoidance at very early stages of development (Alfonso, Peyrafort, Cousin & Bégout, 2020; Edenbrow & Croft, 2011; Ibarra-Zatarain, Rey, Boglino, Fatsini & Duncan, 2020; Polverino et al., 2016), while other investigated its consistency along ontogeny (Alfonso et al., 2020; Castanheira et al., 2013; Edenbrow & Croft, 2011; Polverino et al., 2016). Fernandes-Silva, Leite-Ferreira, Menezes and Luchiari (2022) observed that zebrafish separated by the time of egg hatching (“early hatchers” vs. “late hatchers”) show consistent differences in the exploration-avoidance dimensions when tested at both 30 and 120 days post-fertilization, although individual consistency was not assessed. Alfonso et al. (2020), on the other hand, showed consistent differences in exploration-avoidance between contexts but not over age in zebrafish.

From a mechanistic perspective, some attempts have been made to understand the neurobiological bases of exploration-aggression syndromes. Since both anxiety/avoidance and aggression are related to monoamines in both mammals and fish, with opponent effects of serotonin on avoidance and aggression (Maximino et al., 2013; Olivier, 2004; Paul et al., 2014), these neurotransmitters would naturally be the place to start research. Abbey-Lee, Kreshchenko, Fernandez Sala, Petkova and Løvlie (2019) found that chronic treatment of sticklebacks with fluoxetine decreased the latency to enter a novel area, but did not affect aggressive display; treatment with ropinirole, a nonselective dopamine receptor agonist, decreased both the latency to explore the environment and aggressive displays. Moreover, the expression of stress-related receptor genes (*NR3C1* and *NR3C2*) and a dopamine receptor gene (*DRD1B*) was predictors of individual differences in aggression and sociability (Abbey-Lee et al., 2019). Thus, at least in the experimental contexts proposed by Abbey-Lee et al. (2019), dopaminergic signaling and glucocorticoid hormones appear to be more related than serotonin to the aggression-exploration syndrome.

A mutant strain of zebrafish, *spiegeldanio*, has been shown to carry a mutation in the *fgfr1a* gene, which encodes the fibroblast growth factor receptor 1a. Zebrafish from the *spiegeldanio* strain show increased aggressiveness (tested by the mirror-induced aggression test), as well as increased exploration (measured by the light/dark test, the novel object test, and the latency to fully explore a t-maze) (Norton et al., 2011). *Spiegeldanio* also showed higher exploration than an F1 population derived from wild-caught zebrafish, but not in relation to animals from the AB line (Mustafa, Roman & Winberg, 2019). While *spiegeldanio* showed increased expression of the serotonin transporter *slc6a4a* in the superior raphe, treatment with fluoxetine did not rescue the exploration-aggression syndrome of these mutants. These animals also showed reduced brain histamine levels, and treatment with tacrine, a drug which blocks histamine metabolism, rescued the phenotype (Norton et al., 2011), thus suggesting that histaminergic signaling is responsible for the aggression-exploration syndrome of *spiegeldanio*. Combined with the results from Abbey-Lee et al. (2019), the data from *spiegeldanio* suggest that dopamine and histamine, but not serotonin, are involved in the exploration-aggression syndrome of fish. Nonetheless, it is important to understand that, in both cases, these syndromes have been described at the *population* level, but not at the individual level, and therefore, caution must be taken in interpreting these results.

### 2.3 Activity

A third dimension of fish personality that has consistently been described is *activity*. While activity levels certainly are a confounding factor to measure boldness and exploration (Conrad et al., 2011), sufficient consistency in individual differences in activity levels was found to suggest activity as a personality dimension in itself. Individual variation in activity levels is assessed in multiple tests as a control variable, but consistent individual differences are observed as well. Usually, activity levels are measured as distance traveled (swum) or swimming speed, but measures such as time budgets for specific activities have also been used (Conrad et al., 2011).

There is evidence for activity-boldness syndromes at the population level on various species. Using predator inspection as a surrogate for boldness, Moretz, Martins and Robison (2007) showed positive correlations between activity levels and boldness across one wild-derived and two laboratory-derived populations. Dingemanse et al. (2007) analyzed twelve stickleback populations and found positive correlations between activity, exploration, and aggressiveness only in those populations that were raised in large ponds where piscivorous predators were present. An activity-sociality-exploration syndrome has also been described in sticklebacks, in which an individual’s propensity to stay near others was negatively related to swim speed across tests, and predicted spatial positioning and leadership within groups (Jolles, Boogert, Sridhar, Couzin & Manica, 2017). However, from a mechanistic point of view, studies on activity-boldness and activity-sociality syndromes are absent.

From an ecological perspective, activity levels are of interest because they are correlated with a general metabolic response (Biro & Stamps, 2010; Careau, Thomas, Humphries & Réale, 2008; Nespolo & Franco, 2007). As a result, correlations between activity levels and other personality dimensions can reflect time budget conflicts (e.g., time spent feeding vs. time occupying a refuge), or activity levels can directly reflect metabolism and therefore a constraint on the execution of other tasks (Sih, Bell, Johnson, et al., 2004). Thus, syndromes that involve activity are likely to also be related to metabolism. One theory to explain these correlations is the so-called “pace-of-life syndrome” theory (Réale et al., 2010). This hypothesis states that closely related species or populations occupying different ecological niches are likely to show population-level differences in behavioral traits, as well as in a range of physiological variables. The correlations between these behavioral and physiological traits suggest a co-evolution based on the particular life-history characteristics that are evoked by these niches. Binder et al. (2016) showed that exploration-avoidance is associated with metabolism in the bluegill sunfish (*Lepomis macrochirus*), with individuals with higher exploration (assessed by the emergence test) showing higher maximum metabolic rates and metabolic scopes for activity, but not basal aerobic or anaerobic metabolism.

### 2.4 Aggressiveness

Aggressive behavior has long been a mainstay of ethology, given its ecological relevance in conspecific competition, territory defense, or offspring protection (Réale et al., 2007). While individual variation in aggressiveness has been observed in different situations and species, in the context of personality research, this trait is usually studied in correlation with other dimensions (i.e., a behavioral syndrome)(Conrad et al., 2011; Réale et al., 2007; Toms et al., 2010). This focus on these correlations does not mean, however, that individual differences in aggressiveness were not observed; for example, the seminal study of Huntingford (1976) described individual differences in stickleback aggression.

As is the case with most social behaviors, context-dependent plasticity is very common in the case of aggressiveness (Oliveira, 2012). In a male subpopulation of tropical beau gregory damselfish *Stegastes leucostictus*, aggression levels are individually consistent only in lower-quality breeding sites, as individuals who are transferred to (artificial) higher-quality breeding sites lose this consistency: all individuals become highly aggressive in defending these sites (Snekser, Leese, Ganim & Itzkowitz, 2009). This context-dependent social plasticity is crucial for the ecological and evolutionary consequences of personality dimensions and behavioral syndromes (Luttbeg & Sih, 2010), but has not commonly been addressed in the field of fish personality from the point of view of neuroscience.

Several syndromes have been proposed with aggression (Fig. 1). The aforementioned boldness-aggression syndromes were the first described across species (Bell, 2005; Bell & Sih, 2007; Huntingford, 1976; Moretz et al., 2007), with an activity component sometimes being identified as well (Bell & Stamps, 2004; Dingemanse et al., 2007). Other studies identified an aggression-exploration syndrome, despite calling these correlations an “aggression-boldness syndrome” (Norton et al., 2011; Norton & Bally-Cuif, 2012). Metcalfe and Thorpe (1992) found that, in Atlantic salmon (*Salmo salar*), earlier-feeding fry were dominant over their later-feeding siblings, leading to an increased probability of early-feeding fish migrating to sea 1 year earlier than their siblings. Similarly, in juvenile Atlantic salmon, a positive correlation between social status and standard metabolic rate was found, an effect that impacts the outcome of aggressive encounters (Metcalfe, Taylor & Thorpe., 1995).

The aforementioned *spiegeldanio* zebrafish mutant sheds some light on the aggression-exploration syndrome (Mustafa, Roman, et al., 2019; Mustafa, Thörnqvist, Roman, & Winberg, 2019; Norton et al., 2011; Norton & Bally-Cuif, 2012). As described above, *spiegeldanio* show increased aggressiveness in a mirror test (Mustafa, Thörnqvist, et al., 2019; Norton et al., 2011), an effect that has been attributed to population-level differences in histaminergic signaling; this aggressiveness does not translate into more success in dyadic fights, however, as *fgfr1a* mutant fish did not have any advantage in fights for social dominance, and agonistic behavior of these mutants did not differ from that of AB fish during dyadic interactions (Mustafa, Thörnqvist, et al., 2019). Thus, the aggression-exploration syndrome of *fgfr1a* mutants is not associated with social plasticity, the ability to rapidly switch between behaviors in response to changing social conditions (Taborsky & Oliveira, 2012). Interestingly, social plasticity has been associated with monoamines and nonapeptides, but not yet with histaminergic signaling (Maruska, Soares, Lima-Maximino, Henrique de Siqueira-Silva & Maximino, 2019).

The neuroendocrine hypothalamus-pituitary-interrenal axis (the functional analog of the hypothalamus-pituitary-adrenal axis of mammals) has been implicated in individual variation in aggressiveness. In sticklebacks, within-population variation in aggressiveness is negatively correlated with cortisol levels (Aubin-Horth et al., 2012). Interestingly, a boldness-aggression syndrome has been identified in this population, and both dimensions are positively correlated with brain expression of glucocorticoid receptors (Aubin-Horth et al., 2012). It is possible that the interplay between glucocorticoids and monoamines conjoins these syndromes.

### 2.5 Sociability

Sociability includes any interaction between two or more individuals, which can be positive (i. e. shoaling or cooperation) or negative (i. e. social avoidance or agonistic encounters). Studies using multidimensional statistics suggest that aggressiveness and sociability are separate personality dimensions in fish (Réale et al., 2007; Toms & Echevarria, 2014). Social behavior varies between species, including large groups of fish forming a shoal to interactions between two individuals, between male and females aiming to breed or even between opponents fighting for resources (Maruska et al., 2019). An important element in social behavior is communication; thus, sociality requires that individuals are exchanging information between them, not only sharing the same space.

Several adaptive functions of sociability can be pointed out, such as collective defense, collective searching for food patches, and mating (Taborsky & Oliveira, 2012). Alliances established for territory and group defense are important features that guarantee survival and increase group safety in situations such as foraging in groups (Stenberg & Persson, 2006) and caring for the young (Amundsen, 2003; Bender, Heg-bachar, Oliveira, Canario & Taborsky, 2008). Other benefits of social groups such as shoals may include vigilance and threat signaling by some individuals in favor of group survival (Clark & Dukas, 1994), dilution of risk (Ioannou, Bartumeus, Krause & Ruxton, 2011), and confusion effect or coordinated evasion that reduces predation (Krakauer, 1995). Moreover, shoaling brings advantages in foraging when hunting in groups increases prey capture (Hintz & Lonzarich, 2018). In this case, the size of the group plays a key role in success, as food will be shared with all individuals. Thus, a trade-off exists between the number of individuals foraging together and the competition for the resource (Rieucau, Fernö, Ioannou & Handegard, 2015).

Other instances of social behavior, such as social recognition (Silveira, Silva, Ferreira & Luchiari, 2020) and cooperation (Pimentel et al., 2019, 2021), have been observed in fish. Nonetheless, most of the research on sociability as a personality dimension focuses on shoaling. In sticklebacks, bolder individuals engage in fewer social interactions than shy individuals, but create more diverse social networks (Pike et al., 2008). In guppies (*Poecilia reticulata*), shoaling tendency and boldness (both measured in the laboratory) predict the strength of a social network assessed in the field, with bolder individuals showing weaker social ties than shy individuals (Croft et al., 2009). As is the case with guppies and sticklebacks, boldness-sociability syndromes were also described in zebrafish at the population level, with a negative correlation between these dimensions (Moretz et al., 2007).

Many different mechanisms have been studied in relation to sociability in general. Sexual hormones represent interesting starting points, as steroids have been shown to impact sociability, aggressiveness, boldness, and exploration by themselves (Bender et al., 2008; Diotel et al., 2011; Dzieweczynski, Eklund & Rowland, 2006; Ogawa, Pfaff & Parhar, 2021). Nonapeptides (isotocin-like and vasotocin-like) have also been implicated in those dimensions separately (Kawabata, Hiraki, Takeuchi & Okubo, 2012; Larson, O’Malley & Melloni, 2006; Rose & Moore, 2002; Santangelo & Bass, 2010). Monoamines have also been implicated in both sociability and stress (Soares et al., 2018). Nonetheless, these mechanisms have not yet been investigated in depth as mechanisms for the *correlations* between these dimensions (i.e., behavioral syndromes).

## 3. The use of fish personality in anxiety research

The relationship between personality and psychopathology has been observed extensively in the history of the field. Theoretical models of personality (e.g., RST or Clark’s negative affect-positive affect-disinhibition model) have been developed that incorporate explicit references to psychopathology while simultaneously describing individual differences that can be understood as a continuum between “normal” and “pathological” behavior. While most of the work in fish personality has focused on describing either proximal mechanisms or evolutionary causes of individual differences, the amount of work in the field, as well as the continued appeal to use fish organisms in neuroscience research, can contribute to understanding the relationships between personality, brain mechanisms, and psychopathology. In what follows, we discuss how the five-dimensional model of boldness-exploration-activity-aggressiveness-sociality can be compared to models such as RST (and the difficulties of doing so).

Fish are increasingly being used as model organisms in behavioral neuroscience and experimental psychopathology. A full review of the methods and models that have been proposed using fish (especially *D. rerio*) fall beyond the scope of this article (but see Fontana et al., 2019; Abreu et al., 2020; War, Surendra, Paul, Sharma, Suresh & Manjunatha, 2022, for recent reviews). Nonetheless, extensive work has been done on behavioral models of anxiety-like behavior in zebrafish (War et al., 2022), including the now widely used novel tank test and the light-dark test (Maximino et al., 2012). While potential points of contact have been proposed between research on zebrafish anxiety-like behavior and personality (e.g., Maximino et al., 2012), the full potential has not yet been reached.

The usefulness of fish models in personality neuroscience lies in producing an eco-ethological and evolutionary framework for personality research in general. As discussed in the present work, a lot of research using fish in this field focused on adaptive mechanisms for the evolution of personality differences across populations, as well as on the contexts which sustain these differences in currently existing populations (Conrad et al., 2011; Réale et al., 2007; Weiss & Adams, 2013). However, one difficulty remains: how does the five-dimension model of boldness/exploration/aggressiveness/sociability/activity map to models which are currently favored in non-fish research?

To understand the origin of this issue, one must return to the meta-theoretical considerations discussed at the beginning of this article. In order to provide an evolutionary framework to understand individual variation, population-level differences, and behavioral syndromes in fish, early researchers began with a bottom-up approach, describing exhaustively behavioral differences across contexts in different species. These descriptions eventually coalesced into a common vocabulary that was adopted by investigators that were researching other taxa, including mammals (Whitham & Washburn, 2017). This common vocabulary informed experiments and field observations, effectively initiating a top-down approach: researchers were now no longer cataloging behavioral variation, but doing so with the framework of the five-dimension model in mind. This led to observations on variations in a wide range of species and higher taxa, but distanced animal personality research from other models that are usually applied to common model organisms in neuroscience (mainly rodents and primates).

One of these theories is RST, which has been developed from Jeffrey Gray’s (Gray & McNaughton, 2003) neuropsychology of anxiety (Corr, 2002, 2004; Corr & Perkins, 2006). RST views significant affective events as either positive or negative, postulating three interacting systems that process these events and control behavioral responses to them: the fight-flight-freeze system (FFFS), which mediates reactions to all aversive stimuli, with the associated emotion of fear; the behavioral approach system (BAS), which mediates reactions to all appetitive stimuli, with the associated emotion of anticipatory pleasure; and the behavioral inhibition system, responsible to solve conflicts between approach (BAS) and avoidance (FFFS). Thus, the three systems are associated with different emotions and also represent separate reinforcement sensitivities. These modules are associated with hierarchical brain systems which – at least in the case of the FFFS and the BAS – are highly conserved across vertebrates, including fish (do Carmo Silva, Lima-Maximino & Maximino, 2018; O’Connell & Hofmann, 2012). Individual differences in the overall functioning of these systems are associated with fear-proneness and avoidance (FFFS); optimism, reward orientation, and impulsivity (BAS); and a combination of worry-proneness and anxiety (BIS) (Corr & Perkins, 2006). Thus, normal variation in personality would mean variation in the sensitivity in either of the separate modules and/or general modulatory (e.g., monoamines) influences on the overall system (Corr & McNaughton, 2012). It is important to understand that the general personality consequences of individual variation in the sensitivities of these systems are not the result of activity in a single system, but rather the joint sensitivities of the systems (Corr, 2004; Gray & McNaughton, 2003).

Attempts have been made to correlate aspects of RST with other psychobiological theories of personality, including Eysenck’s (1967) model: Eysenck’s Extraversion and Neuroticism dimensions would be derivative factors of punishment and reward sensitivities, with Extraversion reflecting the balance between both sensitivities and Neuroticism their joint strengths (Corr, 2004). Empirical analyses of the relationship between RST and the five-factor model (“Big Five”) were also made in human subjects, suggesting that Sensitivity to Punishment is positively associated with Neuroticism and Agreeableness and negatively associated with Extraversion, Openness, and Conscientiousness; in contrast, Sensitivity to Reward (SR) was positively associated with Extraversion and Neuroticism, and negatively associated with Agreeableness and Conscientiousness (Mitchell et al., 2007).

Likewise, we have previously attempted to map the five-dimension model of fish personality onto RST (Maximino et al., 2012). We proposed that the shyness-boldness and exploration-avoidance axes could be understood as orthogonal to each other and resultant from individual variation in the joint sensitivities of reward and punishment. Thus, individuals higher on exploration and/or boldness would be more sensitive to rewards, while individuals in the opposite ends of the dimensions (higher on avoidance and/or shyness) would be more sensitive to punishment. Specifically, we predicted that, since the FFFS is more related to fear-proneness and avoidance, it would be more directly involved with shyness, while the BIS would be more involved with avoidance. Importantly, both dimensions are also influenced by reward sensitivity and, therefore, by the BAS.

It is currently not clear how other dimensions of fish personality could map on the other components of RST. One of the possible reasons is that RST proposes general systems that are well-suited to explain and model psychobiological processes underlying anxiety, but are too nonspecific to understand other important factors. For example, the BAS represents a general energizing effect that is not confounded with general arousal, and therefore unlikely to represent the activity dimension fully; moreover, while social decision-making certainly involves evaluating stimulus salience – therefore including and interacting with brain circuits involved in reward processing (O’Connell & Hofmann, 2011, 2012) – there are specific regions of the social brain network that are responsive to social stimuli, and thus, the BAS could not fully represent the sociability dimension as well. It is likely that aggressiveness, sociability, and activity are indirectly related to the RST systems, but other aspects of personality need to be invoked to fully translate RST and the five-dimensional model.

Indeed, as Smillie, Pickering and Jackson (2006) suggested, “[e]xplanation of personality is a compelling by-product of RST, but a by-product nonetheless” (p. 321). RST was primarily concerned not with “anxiety” and “impulsivity” as descriptive personality dimensions, but as spectra of disorders and dispositions. Depue and Collins (1999) identify Gray’s (1981) critique of Eysenck’s arousal-activation model of personality as the beginning of RST as a personality theory that investigated the relationship between personality traits and “basic” processes of motivation and emotion.

Trying to harmonize RST and the five-dimensional model of fish personality is tempting because RST was developed strongly on rodent data, and therefore could “bridge” the translation of both human and fish data. Nonetheless, it is now clear that RST was never intended as a “complete” model of personality. To the best of our knowledge, no attempts have been made to map fish data and the Big Five model; it could be argued that shyness-boldness could conceptually map to Neuroticism, exploration-avoidance could conceptually map to Agreeableness, activity to Conscientiousness, aggressiveness to Extraversion, and sociability to Openness/Intellect. But it could be similarly argued that sociability should map to Extraversion, for example. The difficulty, again, lies at the meta-theoretical level and can be solved partially by addressing that level in conjunction with further empirical research.

One of the difficulties in translating most of the theoretical models of personality developed to understand human data (or, at best, mammalian data) is that direct analogies of behavior between species might not be straightforward or applicable (Trofimova et al., 2022). In part, this is due to over-reliance on looking for links between behavioral traits (i.e., “dimensions” in the sense used in this paper) and morphophysiological traits, especially in the rodent literature (McNaughton & Corr, 2022). The studies reviewed in the present paper provide an evolutionary and comparative context for that and also tend to focus on the relationship between variation in a single behavioral dimension and variation in one (or a handful) biomarker or physiological trait. Some progress has been made in the direction of analyzing how biological variation is related to variation in the *correlation* between dimensions (a systems/domain interplay approach; Kalueff, Ren-Patterson, LaPorte & Murphy, 2008), and, although many methodological and meta-theoretical issues remain, the current context – while not directly and easily translated to mammalian personality – offers great promise as comparative research. De Young (2010), for example, suggested that the traits in the Big Five model can be hierarchically organized in meta-traits of stability and plasticity and that these meta-traits are related to serotonergic and dopaminergic neurotransmission, respectively. While this is still speculative, this could represent a way of simultaneously looking for behavioral variation and neural basis while maintaining the meta-theoretical concerns at close. As can be seen in Table 1, for example, serotonin, dopamine, and histamine appear to participate in almost all dimensions, which can have several explanations: receptor-specific functions of these neurotransmitters, a participation of the neurotransmitter in the pleiotropic modulation of different personality dimensions (and therefore of their correlation at the individual level), or lack of specificity are possible hypotheses.

## 4. Conclusion

The present article reviewed research on fish personality dimensions, presenting a five-dimensional model and some of the caveats and limitations of the field as a whole. Using fish in personality research can contribute to understanding neurobehavioral correlates of personality; the extensive use of these animals to understand the evolutionary basis of personality holds great promise in providing a comparative, ecological, and evolutionary perspective for personality neuroscience. There are many meta-theoretical issues to be solved in the field, including how one defines personality; how different dimensions are measured; what is the unit of analysis; and how to best approach the identification of dimensions (i.e., through a bottom-up or a top-down approach, or a serial combination of both). These issues underline the importance of coherent initiatives in understanding and defining personality and impact the ability of the field to produce mechanistic research. For example, the interesting work on *spiegeldanio* (Norton et al., 2011) reveals promising mechanisms, but differences were observed at the population level, not the individual level, and, while the authors claim to have observed a behavioral syndrome, the correlation between behaviors within populations was not assessed. As a result, the role of histaminergic signaling in an aggression-exploration syndrome remains an interesting theoretical possibility that needs to be further assessed using the tools of personality research.

While the field certainly needs to progress further, with researchers that are seeking proximal mechanisms needing to have a deeper understanding and contact with the ethological-ecological literature, and vice-versa, personality neuroscientists in general can also benefit from fish research. This is especially true given the extensive ethological research that attempted to define the ecological and evolutionary causes of individual differences. The apparent incompatibility between the five-dimensional model and other models of non-human personality may seem like an obstacle, but researchers from both the rodent/primate field and fish researchers can collaborate to harmonize datasets, explicitly stating the meta-theoretical issues that are usually left implicit. Thus, work is needed both within the field of fish personality and in concert with researchers working with rodents and primates to better understand continuities and discontinuities in vertebrate personality neuroscience.
